# Miscellaney

**Published:** 1897-01

**Authors:** 


### Miscellany.

Visiting Graduate Nurses.—Misses Hollywood and Torrey, graduate nurses, announce that they will visit and care for patients at the following rates: One or two hours, 50 cents per hour; each additional hour, 25 cents; remain with patient twenty-four hours, $3.00; remain with patient all day, twelve hours, $2.00; remain with patient all night, twelve hours, $2.50; prepare patient for surgical operation and assist, 50 cents per hour. *Obstetrical Cases.*—First six hours, or less, $2.00; each additional hour, 25 cents; if detained twenty-four hours, $5.00; care of patient and infant, two hours daily, $1.00. Prepare remains for burial, $5.00. Terms, strictly cash. Residence, "The Oxford," 432 Pearl street. Telephone, Tupper 613.

This kind of service may prove a great convenience to both physicians and patients in cases where it is inconvenient or unnecessary to have nurses for longer periods.

